# Optimisation of Negative Fixed Charge Based Edge Termination for Vertical GaN Schottky Devices

**DOI:** 10.3390/mi15060719

**Published:** 2024-05-29

**Authors:** Vishwajeet Maurya, Daniel Alquier, Mohammed El Amrani, Matthew Charles, Julien Buckley

**Affiliations:** 1 CEA, Leti, Université Grenoble Alpes, 38000 Grenoble, France; 2 GREMAN UMR 7347, Université de Tours, CNRS, INSA Centre Val de Loire, 37071 Tours, France

**Keywords:** gallium nitride, vertical Schottky-diodes, edge termination, TCAD modelling

## Abstract

This study focuses on the impact of negative fixed charge, achieved through fluorine (F) implantation, on breakdown voltage (BV) enhancement in vertical GaN Schottky diodes. Several device and implant-related parameters are examined using Synopsys Sentaurus TCAD simulations in order to determine the optimum fixed negative charge concentration required to achieve the highest BV. The simulated structure consisted of a Schottky diode with a box consisting of negative fixed charges to achieve the edge termination of the Schottky device. An empirical equation is proposed to determine the optimum fixed charge concentration for the highest BV based on depth. The simulation also considered implantation profiles derived from SIMS data from an actual device implanted with multi-energy and multi-dose F. It is demonstrated that the BV has a similar dependence on the key parameters like in the box profile. In summary, this work provides valuable insights into optimizing edge termination techniques using negative fixed charge for improved BV in vertical GaN power devices.

## 1. Introduction

Gallium nitride (GaN) offers superior physical properties than silicon (Si) and silicon carbide (SiC) such as large bandgap, high critical breakdown field and high electron mobility [1] necessary for high voltage and high-frequency operations. Lateral GaN-on-Si devices have been commercialized up to 900 V [2] but they suffer from several issues like charge trapping, lower threshold voltage, current collapse, etc. [3]. With the recent advancements in the growth of high-quality native GaN substrates it is now possible to fabricate vertical GaN-on-GaN devices which can enhance the performance of GaN-based power devices. One key challenge related to the development of such devices is the electric field management. In a real device, the observed breakdown voltage is lower than the theoretical values for vertical GaN devices. This is due to the fact that potential is curled at the edge of the Schottky contact/junction which leads to a high electric field. Under high reverse bias, the electric field at the edge can locally reach the critical electric field that can initiate pre-mature breakdown. Therefore, it becomes extremely important to implement edge termination in power devices which can relieve the potential crowding at the edges. Several edge termination techniques like mesa isolation, field plate (FP) [4,5,6], junction termination extension (JTE) [7,8,9], reduced surface field (RESURF) [10] or performing implantation to form resistive GaN using Ar, He, N, etc. [11,12,13,14,15,16,17] have been demonstrated in vertical GaN devices. Unlike Si or SiC power devices, it is difficult to implement JTE structures in vertical GaN devices, due to the the difficulty in achieving selective p-type doping through magnesium (Mg) implantation. This requires complicated ultra-high pressure and high-temperature annealing [18,19,20,21] and capping layers [22,23] to repair implantation induced lattice damage, activate the implanted Mg and prevent decomposition of GaN at these extreme conditions [24].

Due to difficulty in the activation of implanted Mg, highly electronegative F has been used as an alternative by several research groups to form a region of negative fixed charge, which helps to reduce potential crowding at the contact/junction edge. F implantation has been experimentally confirmed to create a negative fixed charge in GaN. In HEMT it is utilized to deplete the 2DEG under the gate and adjust the threshold voltage ($V_{th}$) to higher values [25,26]. First-principle defect calculations carried out within the density functional theory (DFT) [27,28] indicate that the dominant defect configuration of F is negatively charged interstitial $F_{i}^{-}$ with the lowest formation energy in n-GaN whereas $F_{N}^{2+}$ is the dominant defect in p-GaN. In vertical GaN devices, this property of F has been utilized to improve the BV by spreading the electric field away from the contact edge [29,30,31,32,33].

In this work, we simulate the impact of the negative fixed charge on the enhancement of BV in vertical Schottky diodes through extensive simulations in Synopsys Sentaurus TCAD. Several parameters such as depth, concentration, overlap, etc., have been studied in order to propose a design rule for fixed charge-based edge termination. A rectangular box is defined in the drift layer containing fixed negatively charged traps using an inbuilt Synopsys TCAD trap model. Due to the smaller size of the F ion compared to the GaN lattice parameters, the implanted ion tends to channel. This channeling effect causes the ion implantation to produce a Gaussian-like profile instead of an abrupt box-like profile. To accurately consider this non-abrupt profile, additional simulations were conducted using the F profile obtained from secondary ion mass spectrometry (SIMS) measurements on an actual device.

## 2. Simulation Structure

**Figure 1:**
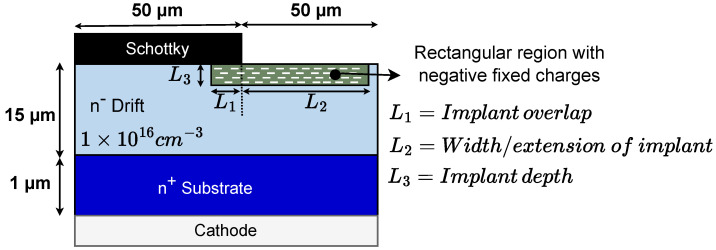
Schematic cross-section of the simulated structure in TCAD. A rectangular box with fixed negative charges has been defined in the structure.

TCAD simulation is performed using the structure as shown in Figure 1. The n-GaN drift layer has a thickness of 15 μm with an n-doping concentration of $1\times 10^{16}$ cm^−3^. A rectangular box is defined in the drift layer consisting of fixed negatively charged traps using the Synopsys trap model.

The following parameters were varied one at a time while keeping the remaining parameters constant to study their impact on the breakdown voltage and electric field distribution:

1. Fixed negative charge concentration.
2. Overlap of the implanted region under the Schottky contact $\left(L_{1}\right)$.
3. Width/extension of the implanted region outside the Schottky contact edge $\left(L_{2}\right)$.
4. Depth of implanted region $\left(L_{3}\right)$.
5. Impact of drift layer doping.

The simulation has been divided into two parts:

1. Using a rectangular region of fixed charge.
2. Using profile obtained from SIMS measurements.

This has been conducted to take into account the Gaussian-like profile of the implanted species. The simulation parameters implemented in TCAD are listed in Table 1. The BV was determined by including impact ionization physics [34] in the simulation and the impact ionization coefficient was taken from [35].

## 3. Simulation Results and Discussions

### 3.1. Box Profile

**Figure 2:**
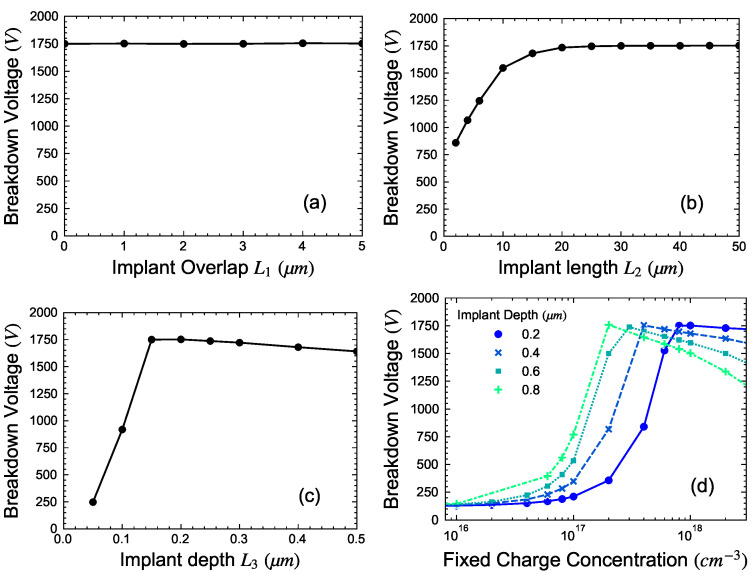
Breakdown voltage vs. (**a**) Overlap (**b**) Implant length (**c**) Implant depth (**d**) Fixed charge concentration.

The influence of different parameters on the BV is plotted in Figure 2.

**Implant overlap $\left(L_{1}\right)$**: As can be seen from Figure 2a, no dependence of the implant overlap under the Schottky contact is observed on the BV. The implant length $L_{2}$, depth $L_{3}$ and fixed charge concentration was taken as 50 μm, 0.2 μm and $1\times 10^{18}$ cm^−3^, respectively.

**Implant extension/width $\left(L_{2}\right)$**: The length of the implanted region was varied by keeping all other parameters constant ($L_{1}$ = 1 μm, $L_{3}$ = 0.2 μm and fixed charge concentration of $1\times 10^{18}$ cm^−3^). As the implant length increases, the BV increases and saturates after a certain length as shown in Figure 2b. This length is approximately equal to the drift layer thickness. For a shorter length, the lateral spreading of the electric field is not sufficient which leads to a lower breakdown due to the equipotential lines crowding at the outer edge of the box.

**Figure 3:**
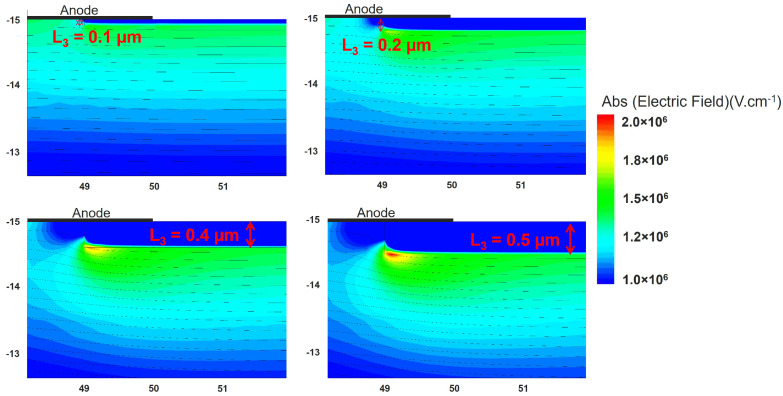
Electric field at −500 V with box depth $(L_{3}$ of 0.1 μm, 0.2 μm, 0.4 μm and 0.5 μm and fixed concentration of $2\times 10^{18}$ cm^−3^). EF is uniformly distributed for a shallower depth and starts to crowd at the vertex as the depth increases.

**Implant depth $\left(L_{3}\right)$ and fixed charge concentration**: The dependence of BV on implant depth $L_{3}$ for a constant fixed charge concentration of $2\times 10^{18}$ cm^−3^ and $L_{2}$ of 50 μm is plotted in Figure 2c. For this simulation, the drift layer doping and thickness were kept constant. A strong dependence on the depth of the box can be observed, with BV first increasing and then decreasing after reaching a maximum value. The 2-D electric field distribution and equipotential lines at −500 V for a fixed charge concentration of $2\times 10^{18}$ cm^−3^ and varying depth are plotted in Figure 3. A clear increase in the electric field at the inner edge of the box can be observed with increasing depth.

**Figure 4:**
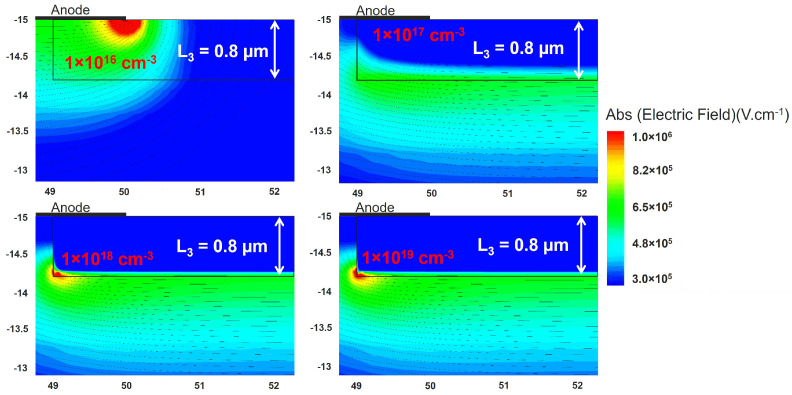
Electric field at −100 V for 0.8 μm depth of box with different fixed charge concentration.

Additional simulations involving an increase in the fixed charge concentration for a fixed box depth indicate the existence of an optimal fixed charge concentration for a given depth. This optimum concentration corresponds to the highest BV, which is equal to the parallel plane BV, as depicted in Figure 2d. The optimum fixed charge concentration decreases with an increase in the thickness of the negative fixed charge region. Moreover, as the implantation depth rises, the reduction in BV becomes more pronounced with increasing concentration. The 2-D electric field profile at −100 V is plotted in Figure 4 for four different concentrations and a depth of 0.8 μm. When the fixed charge concentration is lower than the optimum value, the electric field is crowded at the edge of the contact. As the concentration increases, this peak field is effectively suppressed. For a high concentration, the field lines start to crowd at the vertex of the box leading to a very high electric field and thus reducing the BV.

**Drift layer doping**

**Figure 5:**
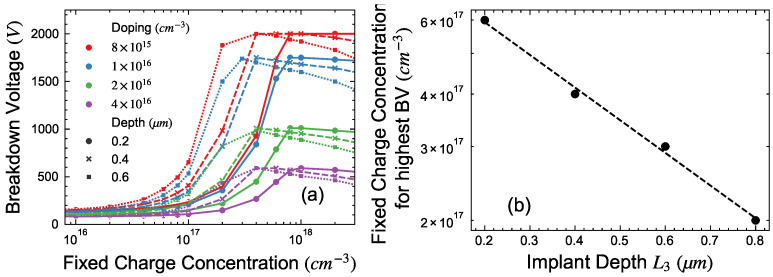
(**a**) BV vs. fixed charge concentration for four different drift layer doping concentrations and three depths. (**b**) Optimum concentration of fixed charge for different depths to achieve highest BV.

The impact of drift layer doping on the optimum fixed charge concentration is plotted in Figure 5a for four different drift layer doping concentrations. The thickness of the drift layer was 15 μm, $L_{1}$ and $L_{2}$ are 1 μm and 50 μm, respectively. As depicted in the plot, the breakdown voltage initially increases with the fixed charge concentration for a given depth of the box, reaching a peak before declining. This optimum fixed charge concentration for a given depth of the box was observed to be independent of the doping concentration in the drift layer and only depends on the depth of the box.

Based on the above results of simulation, an empirical equation can be defined by fitting the fixed charge concentration required for the highest BV and the depth of implant as shown in Figure 5b and can be expressed as:

(1) $$\begin{array}{c} FC=exp^{-Depth\times 1.79+41.28} \end{array}$$

where FC is the fixed charge concentration in cm^−3^ and depth in μm. Using this equation, the optimal implantation dose can be calculated by simply multiplying the concentration by the implantation depth. According to these findings, the determined optimal dose for F implantation is approximately ~$1.6\times 10^{13}$ cm^−2^ irrespective of the drift layer doping and assuming that 100% of the F exists as negative charges.

### 3.2. SIMS Profile

**Figure 6:**
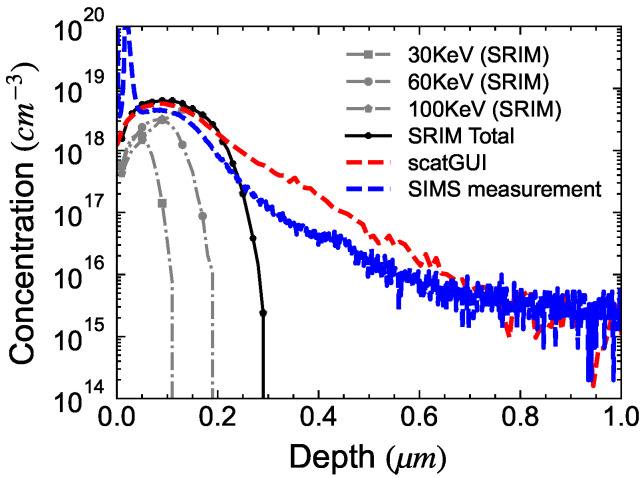
Comparision between Fluorine concentration obtained from SRIM/scatGUI simulation and experimental SIMS.

During an implantation process, ions are accelerated to a specific energy, typically measured in keV, and then bombarded onto a crystal. The acceleration energy determines the depth to which the ions penetrate the crystal lattice. The dose of the implantation (in cm^−2^) governs the concentration of the implanted species. Thus, by controlling both the energy and dose of implantation, one can achieve the desired depth and concentration. Multi-energy implantation is often employed to achieve a more uniform profile. However, achieving an abrupt box profile by implantation in an actual device is challenging due to the phenomenon of ion channeling within the crystal lattice. This discrepancy between the ideal profile and the actual profile can be observed in the Secondary Ion-Mass Spectroscopy (SIMS) profile obtained from samples implanted with F using energies of 30/60/100 keV and doses of $1\times 10^{13}$, $3\times 10^{13}$, and $6\times 10^{13}$ cm^−2^ respectively, as shown in Figure 6. A noticeable difference exists between the simulated profile obtained using the ‘Stopping and Range of Ions in Matter’ (SRIM-2013) software and the measured SIMS profile. The measured profile is also compared with simulations performed using another software called ‘scatGUI’, developed at Hosei University [36]. This software accounts for the channeling of implanted ions and is able to closely reproduce the measured profile.

**Figure 7:**
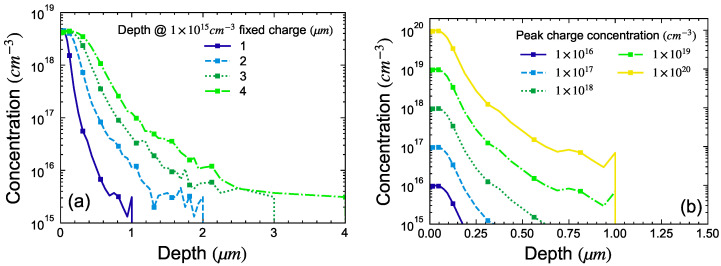
Negative fixed charge profile used in the simulation structure: (**a**) varying depth (**b**) varying concentration.

To account for this deviation from a perfect box-like profile, the TCAD structure was modified by replacing the box of negative fixed charge with the profile extracted from SIMS measurements. This was achieved by directly importing the SIMS concentration-versus-depth profile into a TCAD simulation using a CSV file. Inbuilt TCAD functions were then used to manipulate the SIMS profile in terms of depth and concentration as follows:

1. To emulate different implantation depths, the SIMS profile was scaled in depth while maintaining the peak concentration constant, as shown in Figure 7a. This is analogous to increasing the implant energy.
2. To scale the concentration of fixed charges, the SIMS profile was scaled by the maximum of its absolute value and multiplied by $N_{0}$, as shown in Figure 7b. Here, $N_{0}$ is the peak concentration and was taken between $1\times 10^{16}$ cm^−3^ and $1\times 10^{20}$ cm^−3^. This is analogous to scaling the implantation dose.

**Figure 8:**
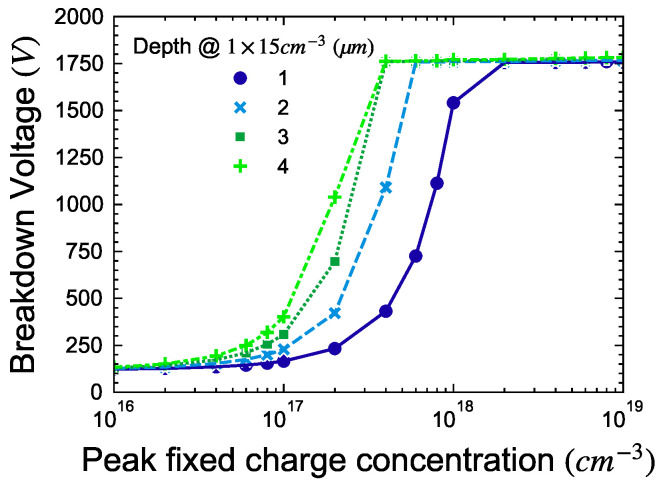
Electric field profile and equi-potential line at −100 V with varying fixed charge concentration.

**Figure 9:**
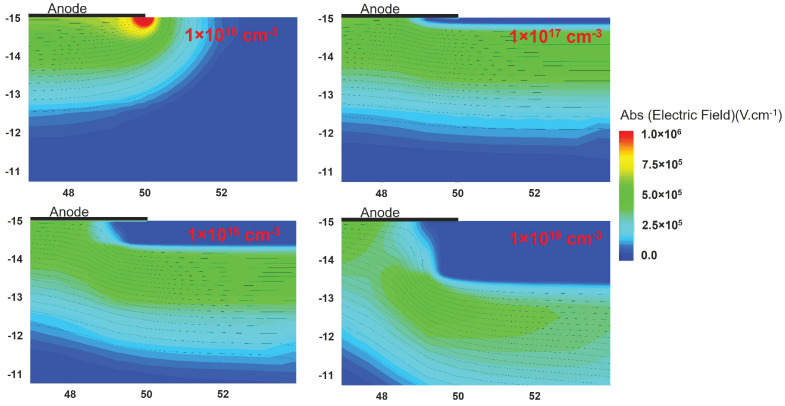
Electric field profile and equipotential line at −100 V with varying fixed charge concentration.

The BV had the same dependence as that of the box profile for the overlap under the contact and implant length, except for the implant depth. As can be observed in Figure 8, the BV plateaus after reaching a maximum which is equal to the parallel plane breakdown voltage for the given drift layer doping and thickness. Figure 9 shows the electric field distribution for four different concentrations (Figure 7b). No electric field crowding inside the drift layer is observed when the concentration of fixed charge is increased, unlike the box profile where the electric field is crowded due to the abrupt junction.

The simulations conducted using the SIMS profile indicate the potential to increase the implant dose beyond the optimal level as determined by the box profile without compromising the BV. This is significant due to the fact that not all the implanted F exists as a negative fixed charge.

The formation energy of F in GaN, calculated through first-principles methods, indicates that in p-type GaN, $F_{N}^{2+}$ is the dominant defect, whereas in n-type GaN, the negatively charged interstitial fluorine $F_{i}^{-}$ is the dominant defect configuration [27,28]. Given that the simulated structure pertains to n-type GaN, $F_{i}^{-}$, with the lowest formation energy, is the primary defect. During the implantation process, lattice damage is inevitable, leading to fluorine occupying positions of N or Ga, in addition to interstitial sites. Research by Wang et al. [37] on the thermal annealing behavior of fluorine-implanted GaN suggests that gallium vacancies ($V_{Ga}$) are the primary vacancy type, which aggregates into clusters post-annealing, and fluorine forms F-vacancy complexes. Molecular dynamics (MD) simulations by [38] have shown that the diffusion potential between interstitial sites and Ga vacancies is the lowest, compared to interstitial sites to nitrogen vacancies or to another interstitial site. Therefore, due to the formation of vacancies and F-vacancy complexes, the number of F at the interstitial site will reduce leading to a reduction in fixed charge density. Our previous study [32] demonstrated that only approximately 10% of the implanted fluorine exists as fixed negative charges. Therefore, it is necessary to reduce vacancy concentration and increase the F concentration in interstitial sites. One possible solution is to perform channeled implantation [39], where the ion beam is directed roughly within ±1° from the 〈0001〉 direction. This method will reduce the vacancy concentration and increase the F concentration in the interstitial site and thus a higher fixed charge concentration.

In the presented simulations, the modulation of implant depth and concentration of fixed charges appears linear when considering an ideal implant box profile. Specifically, a shallower depth necessitates a higher concentration of fixed charges, while a greater depth requires a lower concentration (Figure 5b). Translating this to implantation dose yields a fixed value of ~$1.6\times 10^{13}$ cm^−2^, if 100% of the implanted ions exist as negative charges, forming a perfect box profile. However, real implantation introduces complexities such as ion channeling and defect formation. Consequently, the dose required for ideal conditions may not suffice. Using the SIMS profile, we demonstrated that BV does not degrade when the fixed charge concentration is increased to a high value, unlike the box profile. Therefore, we recommend increasing the total implantation dose beyond the calculated optimum to compensate for these non-ideal effects and ensure effective termination. This approach accounts for non-idealities, including defects and the partial activation of implanted fluorine as negative fixed charges.

In order to implement F implantation-based edge termination, it is necessary to determine the implant profile via simulation first. To have a better estimation of injection depth and diffusion degree, it is advised to use MC simulation software such as scatGUI version 1.37 [36] which considers the channeling effect in GaN crystal lattice rather than SRIM simulation. As can be seen from Figure 6 the simulation performed in scatGUI was able to closely reproduce SIMS measurement and model the channeling effect.

Further simulations are required by using profiles of F and vacancies extracted from software such as scatGUI to model the effects of vacancies more accurately. By incorporating these advanced simulations, we can refine our approach to optimizing ion implantation energy and dose for improved device performance which is a perspective for the presented study.

## 4. Conclusions

In conclusion, we have presented a detailed analysis of the impact of negative fixed charge-based edge termination on the breakdown characteristics of vertical GaN Schottky diodes. Key structural and implantation-related parameters like the depth of implantation, concentration, overlap under the contact and length of implantation are critically analyzed to provide a guideline for dimensioning such edge termination. The simulation results suggested that an empirical equation can be formulated to determine the optimal fixed charge concentration based on the implant depth, facilitating the calculation of an optimal implantation dose. According to the simulations, an optimal dose of approximately ~$1.6\times 10^{13}$ cm^−2^ was identified. Moreover, when considering the SIMS profile, which deviates from a box-like profile, the simulations demonstrated similar trends in breakdown voltage dependence on parameters such as overlap and length. However, unlike the box profile where a reduction in BV was observed after reaching the maximum value, the BV by using SIMS profile plateaus. Electric field distribution simulations indicated that there was no electric field crowding inside the drift layer due to the Gaussian-like profile of negative fixed charge. This allows the implantation dose to be increased beyond the optimum dose as determined by the box profile simulation without compromising the BV. It is important to note that these simulations do not account for the damage (such as vacancies, interstitials, etc.) created during the implantation process. Further investigations are required to comprehensively understand the influence of such damage on the overall performance of vertical GaN SBDs while designing F implantation-based edge termination. Addressing this aspect will contribute to a complete understanding, enhancing the applicability and reliability of the presented guidelines in practical device implementations.

## Figures and Tables

**Table 1:** Key simulation parameters implemented in TCAD.

Parameters	Symbols	Values
GaN bandgap	$E_{g}$	3.44 eV
Relative permittivity	$\varepsilon_{s}$	9.7
Electron mobility	$\mu_{n}$	1200 cm^2^/V·s
Temperature	*T*	300 K

## Data Availability

Data is contained within the article.
